# Low temperature has opposite effects on sex determination in a marine fish at the larval/postlarval and juvenile stages

**DOI:** 10.1002/ece3.6972

**Published:** 2020-11-04

**Authors:** Marc Vandeputte, Frédéric Clota, Bastien Sadoul, Marie‐Odile Blanc, Eva Blondeau‐Bidet, Marie‐Laure Bégout, Xavier Cousin, Benjamin Geffroy

**Affiliations:** ^1^ Université Paris‐Saclay, INRAE, AgroParisTech GABI Jouy‐en‐Josas France; ^2^ MARBEC, Univ. Montpellier, CNRS, Ifremer IRD Palavas‐les‐Flots France

**Keywords:** *Dicentrarchus labrax*, fish, sex ratio, temperature‐dependent sex determination, thermosensitive period, TSD

## Abstract

Temperature‐dependent sex determination (TSD) can be observed in multiple reptile and fish species. It is adaptive when varying environmental conditions advantage either males or females. A good knowledge of the thermosensitive period is key to understand how environmental changes may lead to changes in population sex ratio. Here, by manipulating temperature during development, we confirm that cold temperature (16°C) increases the proportion of fish that develop as females in European sea bass (*Dicentrarchus labrax*) until 56 days posthatching, but show that it has an opposite effect at later stages, with the proportion of males reaching ~90% after 230 days at 16°C. This is the first observation of opposite effects of temperature at different time periods on the sex ratio of a vertebrate. Our results highlight the potential complexity of environmental effects on sex determination.

## Graphical abstract

In the European sea bass, a marine fish species with a polygenic sex‐determination system, we show that exposure to low temperature has opposite effects on sex ratio. Early exposure favors female sex determination, while late exposure favors male sex determination.

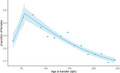

## 1 BACKGROUND

Temperature‐dependent sex determination (TSD) exists in many vertebrate species, including reptiles and teleost fishes (Ospina‐Alvarez & Piferrer, 2008; Pieau, 1996). In TSD species, temperature during early development influences the sex of the animals. There is a multitude of temperature sensitivity patterns, especially in reptiles (Pieau, 1996), but high temperatures mostly bias sex ratios toward males in fishes with TSD (Geffroy & Wedekind, 2020; Ospina‐Alvarez & Piferrer, 2008).

The effect of climate change on species with TSD has long been and remains a subject of great concern (Geffroy & Wedekind, 2020; Janzen, 1994; Mitchell & Janzen, 2010; Ospina‐Alvarez & Piferrer, 2008; Valenzuela et al., 2019), highlighting potential extinction risks, especially in turtles (Jensen et al., 2018; Refsnider & Janzen, 2016; Tomillo et al., 2015). This effect largely depends on the range, but also on the timing of temperature change, that is, on the thermosensitive period. In reptiles, this period corresponds to the first stages of sexual differentiation of the gonads, which happens during incubation (Pieau, 1996). In fishes, it generally happens earlier relative to differentiation (i.e., before gonad differentiation), but after hatching, in the larval and postlarval phases (Baroiller & Guiguen, 1999; Devlin & Nagahama, 2002).

**FIGURE 1:**
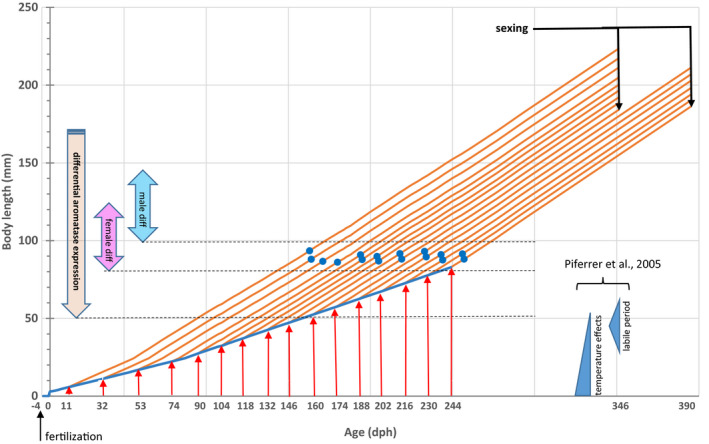
Design of the experiment. Projected body length at 16°C from fertilization (4 days before hatching) to 244 dph (blue line), and projected body length at 21°C (orange lines) of the 16 treatment groups after switching from 16 to 21°C at different ages (indicated by red arrows). Growth is projected from an ad‐hoc empirical growth model. Age and body length at tagging for each group are indicated by blue dots. On the left, arrows indicate the onset of differential expression of gonadal aromatase between males and females (Blázquez et al., 2009) and the size of sea bass when male and female gonads differentiate histologically (Piferrer et al., 2005; Roblin & Bruslé, 1983; Saillant et al., 2003). On the right, shapes indicate the consensus size at which temperature is known to impact sex determination, and the labile period at which exogenous steroids can modify sex ratios, as summarized by Piferrer et al. (2005)

The European sea bass (*Dicentrarchus labrax*), a major fish species for marine fisheries and aquaculture in Europe (Vandeputte et al., 2019), has a sex‐determination system combining larval temperature effects and polygenic genetic variation (Piferrer et al., 2005; Vandeputte et al., 2007; Vandeputte & Piferrer, 2019). The genetic component can be described as polygenic genetic variation for an underlying “sex tendency” which has a heritability in the range 0.34–0.62, with the clear absence of sex chromosomes and even of major sex‐linked QTLs (Faggion et al., 2018; Palaiokostas et al., 2015; Vandeputte et al., 2007). There are little data on the possible interaction between sex tendency and temperature, but their effects seem to be mostly additive, as in a setting with four different families, family rankings for sex ratio were the same at high (21°C) and low (16°C) temperature, while the average proportion of males was significantly increased at high temperature (Anastasiadi et al., 2018). Indeed, high temperature during the larval stage promotes male sex determination, and most studies conform with a model where time spent below 17°C in the larval and postlarval phases (mostly until 55–60 days posthatching—dph, see Figure S1) positively correlates with the proportion of females at the end of the sex‐determination period (Navarro‐Martín et al., 2009; Vandeputte & Piferrer, 2019). For TSD to be adaptive, under the classical Charnov‐Bull model, it is necessary that environmental conditions influence the relative fitness of each sex (Charnov & Bull, 1977), with a “patchy” environment providing advantage to one sex or the other, either in space, within the same breeding season, or in time, across different breeding seasons. In the sea bass, females are larger than males (Saillant et al., 2001), and it is thus likely that larger size has more fitness benefits to females, as generally observed in mass‐spawning species with random pairing (Geffroy & Bardonnet, 2016; Kraak & de Looze, 1993). This leads to an apparent contradiction, as since high temperatures generally promote growth, we would then logically expect a bias toward female sex determination when fish are reared under warm conditions. It was shown on the Atlantic silverside *Menidia menidia* that females were favored by low temperatures because they provide cues regarding the earliness in the season, which thus results in a longer time available for growth in the first year, giving opportunities for the fish to reach a larger size at reproduction on the next spring (Conover, 1984). This may also explain the female‐promoting effect of cold temperatures at the larval and postlarval stages in the sea bass, which was repeatedly seen in many experiments (Figure S1). However, two experiments do not conform to this model. In a first experiment, European sea bass maintained at 13°C from 0 to 342 dph showed a stronger bias toward males (92% males) than fish from the same age kept at 20°C from 15 dph onwards (67% males, Saillant et al., 2002). This observation suggests that low temperature during the juvenile stage (which starts at 50–60 dph, Regner & Dulčić, 1994) may orient sex determination toward males. In a second experiment, European sea bass larvae initially reared at 20–24°C and switched to 15°C at 53 dph were 100% male while fish from the same group kept at 25°C were 87% male (Blázquez et al., 1998). Here again, this suggests that low temperatures during the juvenile stage may bias the sex ratio of a cohort toward males. However, in both experiments, there was a large excess of males even in the “late warm” treatment, likely because the initial cold rearing period, which is expected to favor females, was very short (11 days < 17°C in Saillant et al., 2002) or even absent (Blázquez et al., 1998). In another experiment which tested temperature treatments at 13, 17, or 21°C from 55 to 95 dph, following initial rearing at 17°C, 55% females were obtained at 21°C versus 64% and 65% at 13 and 17°C in a Western Mediterranean population, while in an Eastern Mediterranean population, 55% females were obtained at 13°C, 28% at 17°C, and 37% at 21°C (Mylonas et al., 2005). This indicates that cold temperature can still positively influence female sex determination at that stage. Thus, we hypothesized that there is a first temperature‐sensitive period, in which low temperature orients sex determination toward females, which may extend up to 95 or even 120 dph (up to a body length of 50–55 mm—see Figure 1). Then, with another mechanism, a second thermal sensitive period may exist in sea bass, with cold temperatures favoring males during the early juvenile stage, conforming with a direct negative effect of cold temperature on growth. This second mechanism may start at 50 dph and gain influence over time. It may correspond to the “labile period” mentioned by Piferrer et al. (2005), in which sex can be influenced by treatments with exogenous steroids, and which corresponds to fish in the range 30–70 mm for body length (Figure 1). In any case, when fish reach 80 mm, sex determination is considered complete, as ovaries start to differentiate in females (Figure 1, Roblin & Bruslé, 1983).

In the present experiment, juvenile sea bass reared at 16°C were moved to 21°C at sixteen different time points (starting at 11 dph and ending at 244 dph—Figure 1) This expands the cold period well beyond the usually tested durations, which are generally restricted to the first 70 dph, and sometimes 120 dph (reviewed by Navarro‐Martín et al., 2009). We examined the sex ratio of each of the 16 treatment groups and investigated the underlying molecular mechanisms involved with the expression of two genes with sex‐biased expression (*cyp19a1a* as an early ovarian marker and *gsdf* as an early testis marker) at key periods of the development.

## 2 MATERIAL AND METHODS

### 2.1 Production of fish

The fish population comes from a complete factorial mating of 15 males and 7 females from wild Western Mediterranean origin, performed by artificial fertilization as described in Grima et al. (2010). We used cryopreserved sperm, following Fauvel et al. (1998). Eggs were collected by stripping 72 hr after hormonal stimulation with an injection of 10 μg/kg body weight of luteinizing hormone‐releasing hormone (Sigma, D‐TRP6LHRH). The fertilized eggs, with all families mixed, were incubated at 13°C until 48 hr postfertilization (hpf). Eggs were then evenly dispatched in two tanks of 500 L each, in order to obtain a starting fish density of 50 larvae per liter. Thenceforward, temperature was gradually increased from 13 to 16°C until hatching (61% of eggs hatched, at 96 hpf). Fish were fed on *Artemia* nauplii for 40 days then weaned on a commercial sea bass diet (Marin Start, Le Gouessant).

### 2.2 Temperature treatment and sampling

The major aim of the experiment was to identify the time window at which a temperature change would impact the sex ratio, that is, the thermosensitive period. Thus, we transferred the larvae from 16 to 21°C at sixteen different time points (Figure 1 and Table 1), at 11, 32, 53, 74, 90, 104, 118, 132, 146, 160, 174, 188, 202, 216, 230, and 244 days posthatching (dph). The temperatures were chosen based on the known female‐promoting effect in sea bass of an initial phase at 16°C followed by an increase to 21°C (Vandeputte & Piferrer, 2019). The transfer dates were closer to each other (14 days) after 90 dph, in order to precisely describe sex‐ratio changes in this generally ignored period, while the increase in female proportions with increasing times at 16°C until 90 dph is well documented by previous studies (Figure S1). The last transfer at 244 dph was chosen as the expected stage where fish grown at 16°C would reach the first steps of female sex differentiation, which was later than any known effect of temperature or steroid treatment on sex determination in sea bass (Figure 1 and Piferrer et al., 2005). At that stage, we considered sex was determined, although differentiation, especially in males, may not have started. For each transfer from the initial 500 L tanks (16°C), larvae were randomly fished with a net, counted and placed in a 15 L bucket containing water from the initial tank. The bucket was provided with an oxygen bubbler and floated in a new 110 L tank, in a recirculating system where the temperature was maintained at 21°C. After four hours, when temperatures were equalized, the larvae were gently released in the 110 L tank. Larger numbers of larvae were transferred for the initial switches (Table 1) to try to compensate the large mortality usually observed at early larval stages in sea bass. Survival in the 500 L tanks was 25.0% at 83 dph, which is within the expected range. Survival was much lower for the first group transferred (3.6%, Table 1), indicating a detrimental effect of transfer at this stage. For the other groups, survival increased with age at transfer and reached very high levels (>90%) after 90 dph (Table 1).

**TABLE 1:** Characteristics of the experimental groups. Degree days are calculated with a reference temperature of 10°C. *Estimated values. **Net number = number transferred—number sampled for analyses

Group	Age (dph) at temperature switch	Degree‐days (base 10) at temperature switch	Net** Number of larvae transferred at 21°C	Average body length (mm) at temperature switch	Number remaining at tagging	Survival transfer to tagging (%)	Age(dph) at tagging	Degree‐days (base10) at tagging	Average body length (mm) at tagging	Survival tagging to sexing (%)
1	11	69	6150^a^	5.6	219	3.6%	158	1,670	89.7	98.5%
2	32	195	2100^b^	11.1*	219	25.9%	158	1,566	82.9	98.0%
3	53	325	890^c^	18.1*	218	33.1%	166	1,556	85.4	93.5%
4	74	453	500	25.0*	236	47.2%	175	1,546	82.6	88.1%
5	90	554	300	27.3	250	83.3%	189	1,627	89.3	90.5%
6	104	636	300	27.7	278	92.7%	189	1,564	85.1	89.0%
7	118	722	250	38.3	248	99.2%	199	1,610	88.5	64.0%
8	132	809	250	42.1	228	91.2%	199	1,548	86.8	96.5%
9	146	892	250	48.9	225	90.0%	213	1,640	91.2	69.5%
10	160	977	220	52.6	219	99.5%	213	1,569	84.9	88.5%
11	174	1,060	220	56.8	217	98.6%	228	1,663	91.0	93.0%
12	188	1,147	220	64.2	215	97.7%	228	1,594	84.7	95.0%
13	202	1,231	220	69.1	220	100%	238	1,629	90.0	98.5%
14	216	1,318	220	69.4	218	99.1%	238	1,558	82.3	94.5%
15	230	1,405	220	78.3	220	100%	251	1,637	85.9	92.5%
16	244	1,491	220	82.3	220	100%	251	1,569	82.5	93.5%

Abbreviation: Dph, days posthatching.

^a^ Group 1 fish were 220 at 118 dph (3.6% survival from transfer).

^b^ Group 2 fish were 547 at 118 dph (26.0% survival), readjusted to 220.

^c^ Group 3 fish were 297 at 118 dph (33.4% survival), readjusted to 220.

The sixteen experimental groups were kept at 21°C in separated 110 L tanks until fish reached a minimum length of 8 cm (around 1,600 degree‐days, calculated with a base temperature of 10°C, see Table 1), where sex determination is considered complete with the start of ovary differentiation (Figure 1, Roblin & Bruslé, 1983). At that time, fish were anesthetized (45 mg/L benzocaine) and 200 of them were randomly sampled from a total ranging between 215 and 278 (Table 1). They were individually measured, weighed, and tagged with RFID glass tags (Biolog‐ID, France). They were transferred to 1,500 L tanks and mixed by groups of the three closest temperature switch dates (600 fish in each 1,500 L tank, from groups 1–2–3, 4–5–6, etc…). This was done to save space and is not supposed to impact the sex ratio as fish are larger than 8 cm. At 324 dph, all fish were individually weighed. From tagging to sexing, fish were fed *ad libitum* and temperature averaged 21.4 ± 0.7°C. Survival from tagging to sexing was generally high (>90%) although a few groups experienced significant mortalities (Table 1). Fish were euthanized using benzocaine (150 mg/L) at 346 dph (groups 1–9) or 390 dph (groups 10–16), and sexed by *in situ* gonad examination (Figure 1), or in uncertain cases by microscopic observation of a gonadal squash (Menu et al., 2005). Fish rearing was performed at the Ifremer Plateforme Expérimentale d'Aquaculture (Palavas‐les‐Flots, France), accredited to use and breed laboratory animals (n°C341926) and the project was approved by the Animal Care Committee # 84 COMETHEA under project authorization number APAFIS 10745.

### 2.3 Gene expression analysis

Among genes involved in the sex differentiation cascade in fishes, gonadal aromatase (*cyp19a1a*), encoding the enzyme converting androgen into estrogen, is used as an early ovarian marker (Blázquez et al., 2009; Guiguen et al., 2010) and gonadal soma derived factor (*gsdf*), stimulating spermatogonia proliferation (Shibata et al., 2010), is used as an early testis marker (Geffroy et al., 2016). The expression of these genes was thus studied at specific time points.

Ten larvae were sampled for gene expression studies in the initial tank at 16°C (considered the control protocol) at 75 and 96 dph, and at the same dates ten larvae per tank were also sampled in the 21°C tanks containing fish switched at 32, 53, and 74 dph.

#### 2.3.1 Extraction of total RNA from fish samples

Larvae were individually grinded in MR1 and 1 μl of TEPC following manufacturer instructions (Macherey‐Nagel). We used a KingFisher Flex automatic extraction robot with all reagents provided in the NucleoMag® RNA kit (Macherey‐Nagel) for the RNA extraction of all samples.

#### 2.3.2 Reverse transcription

RNA quantity was assessed by measuring the A260/A280 ratio using the NanoDrop® ND‐1000 V3300 Spectrophotometer (Nanodrop Technology Inc.). Complementary DNAs were synthesized using the GoScript™ Reverse Transcription System kit following manufacturer instructions (Promega) with a starting quantity of 1 μg of total RNA as in (Sadoul et al., 2018). All cDNAs were diluted 25 folds in nuclease‐free water prior to quantitative real‐time PCR (qPCR).

#### 2.3.3 Quantitative real‐time PCR

The *cyp19a1a* primers were taken from the literature (Navarro‐Martín et al., 2011), while *gsdf* primers were designed using Geneious Prime® software and the published (annotated) genome of the European sea bass (Tine et al., 2014). The cDNA product was then sequenced, confirming the identity of both genes. We used the following housekeeping genes: ribosomal protein L13 (*L13*), the Eukaryotic translation elongation factor 1 alpha (*eef1‐alpha*) and the β‐Actin (*actb*) as in Sadoul et al. (2018). Primer sequences and efficiency are given in Table S1. An Echo®525 liquid handling system (Labcyte Inc.) was used to dispense 0.75 μl of SensiFAST ™ SYBR® No‐ROX Kit (Bioline), 0.037 μl of each primer, 0.21 μl of ultra‐pure water, and 0.5 μl of diluted cDNA into a 384‐well reaction plate. Each sample was run in duplicate. The qPCR conditions were as follows: denaturation at 95°C for 2 min, followed by 45 cycles of amplification (95°C, 15 s), hybridization (5 s) and elongation (72°C, 10 s), and a final step at 40°C for 30 s. Primers hybridization temperatures were 64°C for *cyp19a1a* and 60°C for all other genes. Relative levels of gene transcription were obtained using the following equation (*E*^(Ct_ref))/*E*^(Ct_target) with the target gene normalized by the geometric mean of the three housekeeping genes as reference (*L13, actb,* and *eef1‐alpha*), and *E* representing the real efficiency of the standard curve. Note that the mean CT for both genes was relatively high (*cyp19a1a* = 34.3 and *gsdf* = 31.5); this was due to the fact that we used whole larvae (instead of gonads) to assess gene expression, but this approach was previously performed and validated in comparable studies on the subject (Blázquez et al., 2008, 2009).

### 2.4 Statistical analysis

The sex ratio in the 16 treatment groups (0 for male, 1 for female) was analyzed by logistic regression, with age at temperature switch as the explanatory variable, with glm in R (https://www.r‐project.org/). Then, a segmented logistic regression was adjusted to the same data (R package segmented, Muggeo, 2019). As there was a strong statistical support for the existence of a breakpoint, this segmented regression was considered the most appropriate model. Data from the 11 dph switch were removed from the analysis, as the transfer protocol caused a high mortality (>80%) in the days following transfer. Details of this as well as analyses including this time point are given in Appendix S1 and Figure S2.

We also analyzed how sex dimorphism for body weight was affected by the temperature treatments. Body weight at 324 dph was analyzed with an ANCOVA model with sex as factor and age at temperature switch as a regressor, as well as the interaction between both. To account for a possible scale effect, the same analysis was performed on log‐transformed body weight at 324 dph.

For the gene expression analysis, samples with a geometric mean of the CT for the reference genes above the last standard curve point (*i.e.,* cDNA diluted 320 times) were removed. This represents 7 samples (5.5%) on a total of 127 samples. Nonparametric tests (Kruskal–Wallis test followed by Wilcoxon test with Bonferroni correction, considered significant when *p* < .05) were used to compare gene expression within and between (comparing only those at 96 dph) the different temperature protocols.

## 3 RESULTS

**FIGURE 2:**
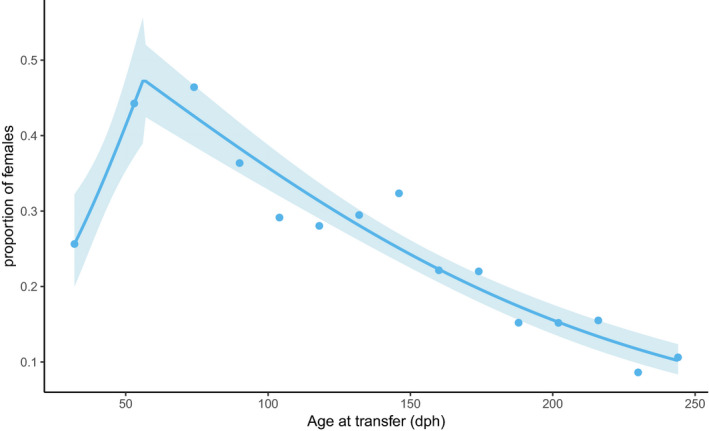
Change in the proportion of females, as a function of age at transfer from 16 to 21°C. Each data point represents a group with *N* = 136–206 (average 177) animals. Regression lines are a segmented logistic regression

There was a general trend to a decrease in the proportion of females with the time spent at 16°C before the switch to 21°C (logistic regression, slope = −0.0079 ± 0.0007, *z* = −11.02, *p* < .001). However, there was strong support for a segmented logistic regression with a breakpoint at 56.2 ± 3.9 dph (*p* < .001, Figure 2). Body weight at 324 dph was strongly negatively affected by age at temperature switch (*F*_1,2659_ = 1,029.3, *p* < .001), by sex, with females being larger than males (*F*_1,2659_ = 124.0, *p* < .001) and by their interaction (*F*_1,2659_ = 13.4, *p* < .001, Figure S3). When log‐transformed body weight was analyzed with the same ANCOVA model, to control for scale effects, age at temperature switch still had a significant effect (slope = −0.0034 ± 0.0002, *F*_1,2659_ = 1,144.4, *p* < .001) as well as sex (females ‐ males = 0.205 ± 0.036, *F*_1,2659_ = 126.0, *p* < .001), but interaction was not significant anymore (*F*_1,2659_ = 0.88, *p* = .34), showing that both sexes were similarly affected by age at temperature switch.

**FIGURE 3:**
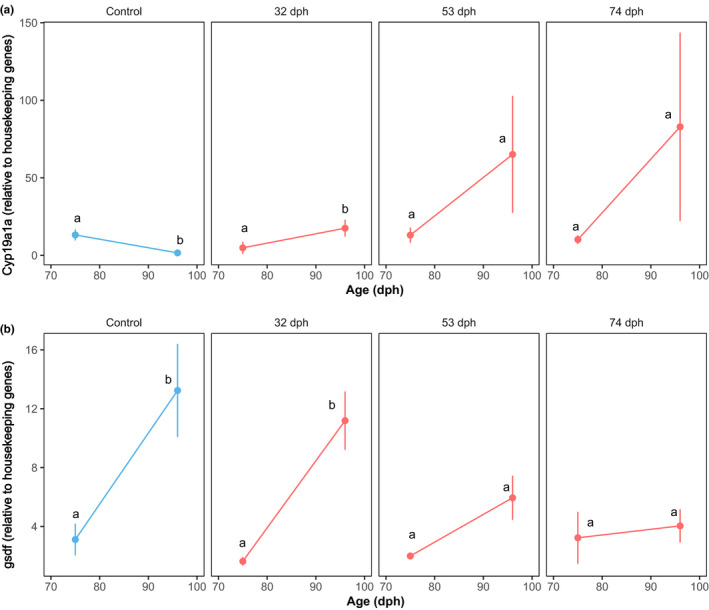
Comparative expression of *cyp19a1a* (a) and *gsdf* (b) at 75 and 96 dph in control fish (16°C) and in fish switched to 21°C at 32, 53 and 74 dph. Mean ± *SEM*; different lower case letters indicate significant differences between ages within treatments

Gonadal aromatase expression (*cyp19a1a*) decreased between 75 (*n* = 9) and 96 (*n* = 7) dph for the fish kept at 16°C throughout the entire experiment (Wilcoxon test, *p* = .008, Figure 3a ‐control), while it increased for fish switched at 32 dph (Wilcoxon test, *p* = .003, 75 dph: *n* = 10 and 96 dph: *n* = 9, Figure 3a). The increase in the other two switch protocols (Figure 3a) was not significant, likely due to a very high variability between samples at 96 dph. When comparing gene expression values between fish sampled at 96 dph, we detected a significant difference only between the control and the group switched at 32 dph (Wilcoxon test, *p* = .003, Control: *n* = 7 and 32 dph switch: *n* = 9).

The expression of *gsdf* increased between 75 and 96 dph in the 16°C control (Wilcoxon test, *p* = .02, 75 dph: *n* = 9 and 96 dph: *n* = 7, Figure 3b) and in the 32 dph switch (Wilcoxon test, *p* < .001, 75 dph: *n* = 10 and 96 dph: *n* = 9, Figure 3b), but not in the 53 and 74 dph switches.

## 4 DISCUSSION

The main result of this experiment is that the time spent at low temperature increases the proportion of females in sea bass, but only until 56–75 dph when further extension of time spent at low temperature produces the opposite effect. The percentage of females in the population reached as low as 10% when the cold rearing lasted more than 230 days. The female‐promoting initial “low‐temperature” period is in agreement with most previous reports (Navarro‐Martín et al., 2009; Vandeputte & Piferrer, 2019) while the second “low‐temperature” period promoting male explains the results obtained by Saillant et al. (2002). This observation is unlikely to be caused by sex‐specific selective mortality of females. As usual during the larval stage, the mortality was high in the initial weeks and sharply decreased thereafter. Mortality rates from transfer to 21°C to tagging (a time at which sex is determined) were low (<10%) for all transfers performed after 104 dph (Table 1) and unlikely to explain the large differences observed in sex ratios. Mortality was not precisely recorded after 83 dph in the large 16°C tanks where fish were sampled to be transferred at 21°C, but no noticeable mortality was seen during the whole experimental period.

This advocates for the existence of two temperature‐sensitive periods in sea bass, with opposite consequences of the temperature change. To our knowledge, this was never observed in any animal species. We know only two published cases of TSD species with two distinct temperature‐sensitive periods, *Alligator mississippiensis* (McCoy et al., 2015) and *Oreochromis niloticus* (Rougeot et al., 2008), but in both cases temperature acts in the same direction on sex ratio in the early and late periods.

Our data bring coherence to the existing sea bass literature on temperature sex determination. First, a consistent pattern for the effect of low temperature at early stages is observed across multiple studies (Figure S1), with the proportion of females increasing when time spent at cold temperature extends up to 60 dph. Then, from 60 to 120 dph, varying patterns were observed, and finally the few data available showed a decreasing proportion of females when cold temperature is continuously applied at later stages. The present experiment clearly shows that a long‐lasting exposure to cold temperature would nullify and even reverse the female‐promoting effect of early cold temperature. When combining male promoting warm initial rearing (20–24°C) with cold temperature starting at 55 dph, it is even possible to reach 100% males in a cohort (Blázquez et al., 1998). While early cold temperature can be interpreted as a signal of earliness in the season, and thus a potential for high growth over the first year, as suggested by Conover (1984), the occurrence of low temperature late in the season depresses growth. Such conditions would then be less favorable to females, which are expected to get fitness benefits from a larger size, as generally observed in mass‐spawning species with random pairing (Kraak & de Looze, 1993), which is the case of the European sea bass (Pickett & Pawson, 1994). Here, we clearly saw that females were larger than males of the same age, whatever the length of the cold period. As expected, fish exposed to long cold rearing were smaller than the others at 324 dph, but although absolute differences in body weight between males and females were smaller when fish were exposed to longer cold periods (significant sex * switch age interaction), proportional differences in body weight were unaffected by the length of the cold treatment (no sex * switch age interaction on log‐transformed body weight).

Fish kept at low temperature until 96 dph showed reduced aromatase (*cyp19a1a*) expression at 96 dph compared with the values at 75 dph (Figure 3a, “control” panel), thus reducing the potential for female differentiation, while aromatase slightly increased in the same time window for fish switched at 32 dph (Figure 3a, “32 dph” panel). An increase in mean aromatase expression was also seen between 75 and 96 dph, for fish switched to 21°C at 53 and 74 dph, although it was not significant due to the high variability of expression at 96 dph, where some individuals showed very high aromatase expression levels (Figure S4), which are typical of future females (Blázquez et al., 2009). One might consider surprising the fact that the level of aromatase in fish kept at 16°C for 96 days is below the level observed for fish switched to 21°C at 32 dph, as this appears contradictory with the fact that there were more females in groups switched to 21°C at 90 or 104 dph (36.4% and 29.1%, respectively) than in groups switched at 32 dph (25.6%). However, the development stage of fish kept at 16°C for 96 days was much less advanced, as they accumulated only 591 day degrees (base 10) versus 904 day degrees at 96 dph for the fish transferred to 21°C at 32 dph. Thus, fish kept at 16°C for 96 days were probably too young to express significant amounts of aromatase, as they were far below the size of 50 mm at which increased expression of aromatase starts in females (Figure 1 and Blázquez et al., 2009).

We detected an opposed pattern for *gsdf*, with an increase in expression at 96 dph in the treatments that biased sex ratio toward males, corresponding to either long cold (Figure 3b, control) or short cold (Figure 3b, 32 dph) treatments, but not to intermediate ones (53 and 74 dph switches). Thus, using both *gsd*f and *cyp19a1a,* we were able to detect a positive effect toward female sex determination of initial cold temperatures, but also a clear trend toward male sex determination for fish kept 96 days at 16°C. This advocates for an effect of late cold temperature not on sex differentiation, through sex reversion of already determined males and females, but really on sex determination. Indeed, the temperature switch was applied to fish of 11–82 mm mean body length (Table 1, Figure 1), thus before the start of sex differentiation which occurs first in females, at a body length of 80–100 mm (Papadaki et al., 2005; Roblin & Bruslé, 1983; Saillant et al., 2003).

A previous study showed that the promoter of aromatase is methylated when fish are exposed to high temperature at the larval and postlarval stages, inhibiting the production of estradiol (Navarro‐Martín et al., 2011). This explains the first pattern, where early exposure to high temperature triggers male sex determination, but not the second, where long‐term exposure to low temperature also results in a higher proportion of males. An increase in temperature at the right time could be required to potentiate the female development pathway. In several fish species, an increase of the number of primordial germ cells (PGC) at a precise time point has been associated with the development of an ovary (Adolfi et al., 2019; Lewis et al., 2008), while artificial PGC depletion during this period generally leads to testis development (Adolfi et al., 2019; Slanchev et al., 2005; Tzung et al., 2015). Increasing temperature at the right moment would be transduced to high growth and activate mitosis in PGC, favoring the female development pathway, but no increase would reflect a “low quality” environmental patch, resulting in testis differentiation, which may still be possible in an initially female‐oriented gonad, as shown in a turtle (Dorizzi et al, 1996).

From an adaptive point of view, early low temperature (in the larval and postlarval stages, before 60 dph or 20 mm body length) can be considered a signal of earliness in the season, and thus as a promise for later growth, as observed in the Atlantic silverside (Conover, 1984). As in sea bass, sex differentiation occurs much later in development (80–100 mm body length), this leaves a considerable time window for temperature to directly influence growth. We thus expected that prolonged cold temperatures would be less favorable to female sex determination, and this is what we observed. This may partly explain why the “good” year classes, which contribute to a large part of catches in the Northern part of the distribution of the species, show a higher proportion of females than the other year classes (Kennedy & Fitzmaurice, 1972; Vandeputte et al., 2012). This brings further complexity in the understanding of the potential effects of climate change in sea bass, as higher early temperatures will bias population sex ratio toward males, while late high temperatures may favor females. As the sea surface temperature warming trend is maximal in June in the Mediterranean, but with a rather clustered distribution (very high in the North, much less in the South and East (Pastor et al., 2018), different consequences can be foreseen for the Western and Eastern Mediterranean sea bass populations. However, a full understanding of this will require further studies to describe the reaction norm of sea bass sex ratio to variations of temperature between 20 and 80 mm body length, as well as to understand the exact physiological mechanisms involved. Additionally, it is also possible that different populations would react differently, as sex‐determination QTLs differ between the main populations of European sea bass (Faggion et al., 2018). An interesting aspect is the observed positive genetic correlation between sex tendency and growth (rA = 0.50–0.69, Faggion et al., 2018; Vandeputte et al., 2007). It would be very interesting to see if the genetic association between growth and female sex determination acts on the first or on the second period of sex determination. Our results are a call for additional research on TSD species that could reveal unexpected patterns of temperature effects on sex. This would especially be relevant to fish, as the general pattern in TSD fish species is that sex determination happens before histological gonadal sex differentiation (although some overlap may exist), leaving ample time for various environmental pressures to apply (Baroiller & Guiguen, 1999; Devlin & Nagahama, 2002).

## CONFLICT OF INTEREST

The authors declare no conflict of interest.

## AUTHOR CONTRIBUTION

**Marc Vandeputte:** Conceptualization (equal); Data curation (equal); Formal analysis (equal); Writing‐original draft (equal); Writing‐review & editing (lead). **Frédéric Clota:** Data curation (equal); Investigation (equal); Writing‐original draft (supporting); Writing‐review & editing (supporting). **Bastien Sadoul:** Data curation (equal); Writing‐review & editing (supporting). **Marie‐Odile Blanc:** Data curation (equal); Writing‐review & editing (supporting). **Eva Blondeau‐Bidet:** Data curation (supporting); Writing‐review & editing (supporting). **Marie‐Laure Bégout:** Funding acquisition (equal); Project administration (equal); Writing‐review & editing (supporting). **Xavier Cousin:** Conceptualization (supporting); Funding acquisition (equal); Project administration (equal); Writing‐review & editing (supporting). **Benjamin Geffroy:** Conceptualization (equal); Data curation (equal); Formal analysis (equal); Writing‐original draft (equal); Writing‐review & editing (supporting).

## Supporting information

Supplementary Material

## Data Availability

Biometric and gene expression data as well as R analysis workflows are available at: Vandeputte, Marc; Clota, Frédéric; Sadoul, Bastien; Blanc, Marie‐Odile; Blondeau‐Bidet, Eva; Bégout, Marie‐Laure; Cousin, Xavier; Geffroy, Benjamin, 2020, “Data for: Low temperature has opposite effects on sex determination in a marine fish at the larval/post‐larval and juvenile stage,” https://doi.org/10.15454/QSMRMX, Portail Data INRAE.
