# Supplementary material for: Low temperature has opposite effects on sex determination in a marine fish at the larval/postlarval and juvenile stages
Source: Ecol Evol. 2020 Nov 4;10(24):13825–35. doi: 10.1002/ece3.6972 (PMC7771145; doi:10.1002/ece3.6972)
Supplement: Supplementary file 1 — Supplementary Material [file ECE3-10-13825-s001.docx]

Supplementary material for :

**Low temperature has opposite effects on sex determination in a marine fish at the larval/post-larval and juvenile stages.**

Marc Vandeputte^1,2*^, Frédéric Clota^1,2^, Bastien Sadoul^2^, Marie-Odile Blanc^2^, Eva Blondeau-Bidet^2^, Marie-Laure Bégout^2^, Xavier Cousin^1,2^, Benjamin Geffroy^2^.

^1^ Université Paris-Saclay, INRAE, AgroParisTech, GABI, 78350 Jouy-en-Josas, France

^2^ MARBEC, Univ. Montpellier, CNRS, Ifremer, IRD, 34250 Palavas-les-Flots, France

**
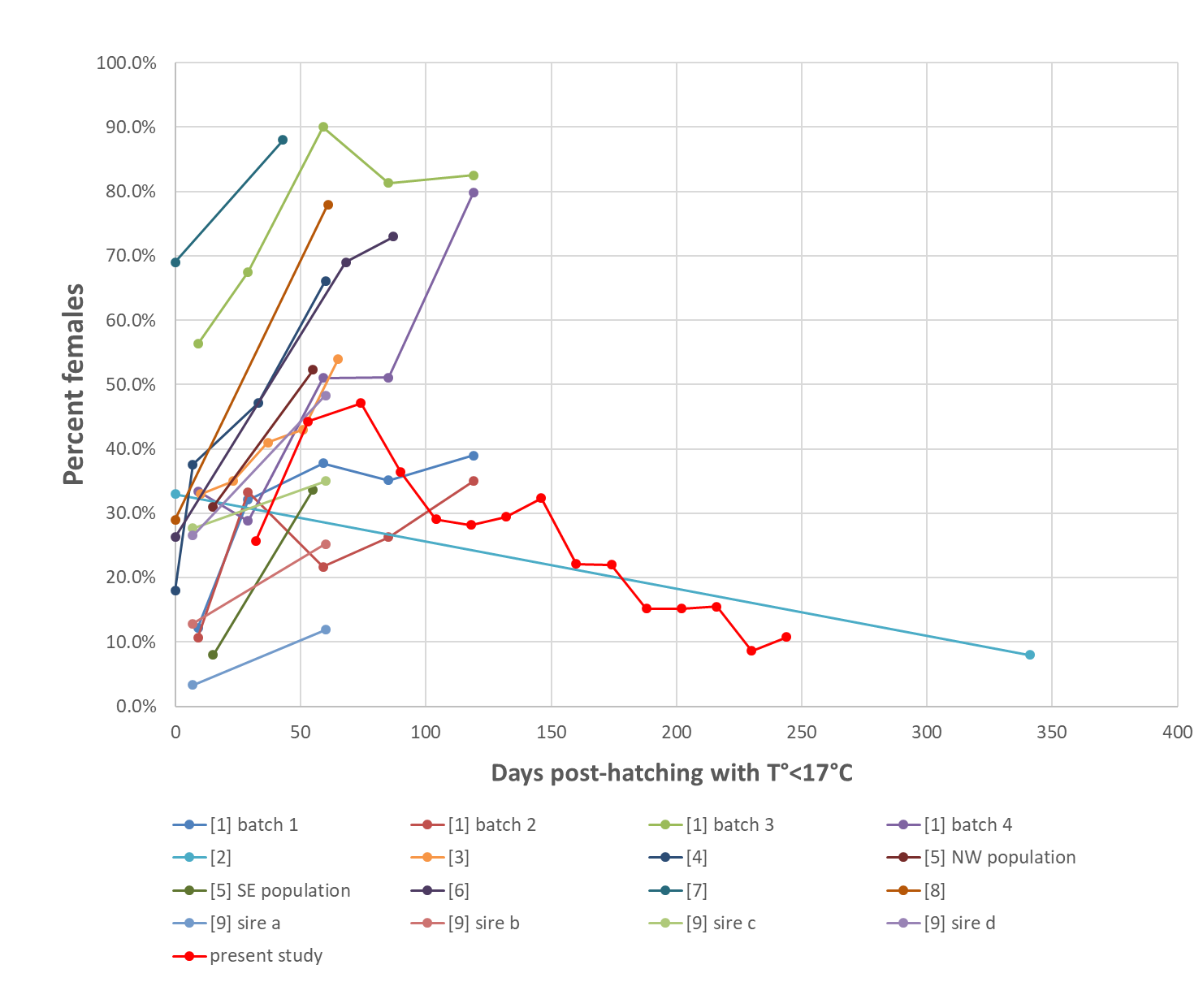
**

**Figure S1:** Effect of the duration of cold (<17°C) rearing after hatching on the percentage of females in European sea bass, in different experiments reported by [1–9] and in the present study. Values from references [1-7] are as reported by [1] in a meta-analysis.

**Table S1:** Forward and reverse primer sequences for the target (*cyp19a1a, gsdf*) and reference (*L13, actb, eef1-alpha*) genes

| Gene | GenBank accession numbers | Primers | Primer sequence 5' to 3' | Efficiency |
| --- | --- | --- | --- | --- |
| *cy19a1a* | DQ177458 | cyp19a1a-F | AGACAGCAGCCCAGGAGTTG | 1.97 |
|  |  | cyp19a1a-R | TGCAGTGAAGTTGATGTCCAGTT |  |
| *gsdf* | DLAgn_00083310 | gsdf-F | TCCATCATCCCACACCAACG | 1.99 |
|  |  | gsdf-R | ATGTTGCCATGTTCACAGCC |  |
| *ef1-*α | AJ866727.1 | ef1-F | AGATGGGCTTGTTCAAGGGA | 2.06 |
|  |  | ef1-R | ACAGTTCCAATACCGCCGA |  |
| *actin* | AY148350.1 | act1-F | CGACCTCACAGACTACCT | 2.05 |
|  |  | act1-R | GCTCGTAACTCTTCTCCA |  |
| *L13* | DLAgn_00023060 | L13-F | TCTGGAGGACTGTCAGGGGCATGC | 2.17 |
|  |  | L13-R | AGACGCACAATCTTGAGAGCAG |  |

**Supplementary text 1:**

Data were initially analyzed with all sixteen transfer times. However, the first transfer was considered potentially problematic because the transfer protocol at 11 dph resulted in a very high mortality in the following days, likely on a size-selective basis (higher survival for the biggest individuals). This mortality is likely higher than 80%, as shown by the very low survival from transfer to tagging in group 1 (3.6%), compared to the general survival of 25% in the 16°C tank from hatching to 118 dph (Table 1). When data were analysed with all sixteen time points, there was a general trend to a decrease in the proportion of females with the time spent at 16°C before the switch to 21°C (logistic regression, P<0.001). There was also a strong support for a segmented logistic regression with a breakpoint at 74 dph (P<0.001, Figure S1) instead of 56 dph in the data without the 11 dph switch (Figure 1).

However, in addition to the fact that this group suffered high initial mortality, the percentage of females in the 11 dph group was high (44% females) and would contradict all previous experiments on the European sea bass, where there always is an increase in the proportion of females as time below 17°C increases, at least until 60 dph (see Figure S1). For these reasons, we decided to discard this data point, although it does not change much the general conclusions (the changes are a break point at 74 vs 56 dph, and a lower positive slope before the breakpoint if the 11 dph point is kept).


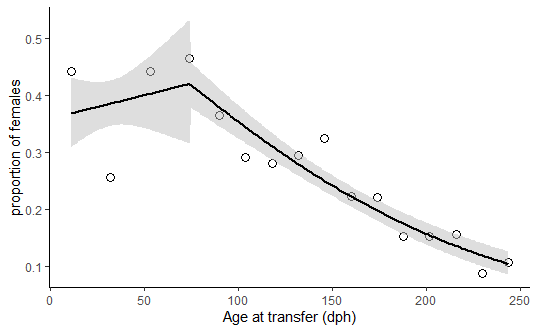


**Figure S2 :** Change in the proportion of females, as a function of age at transfer from 16°C to 21°C. Each data point represents a group with N=136-206 (average 177) animals. Regression lines are a segmented logistic regression. Unlike figure 2 in the main text, this figure includes data from the first time point (11 dph switch) which was removed in Figure 1 as it was considered not to be representative due to high mortality.

**
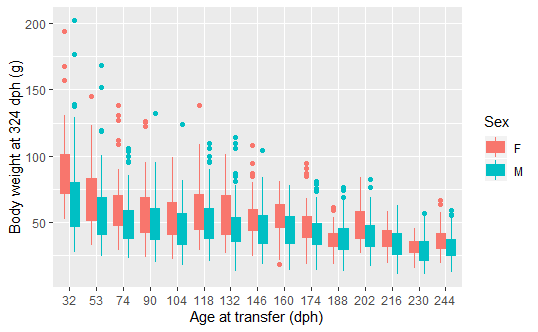
**

**Figure S3 :** Body weight of male and female European sea bass at 324 dph as a function of the age at which temperature was switched from 16 to 21°C.

**Table S2: Base data for figure S1**

| reference | Treatment name | dph T°<17°C | Percent females |
| --- | --- | --- | --- |
| [1] | Batch1 G10 | 9 | 12.2% |
| [1] | Batch1 G30 | 29 | 32.1% |
| [1] | Batch1 G60 | 59 | 37.8% |
| [1] | Batch1 G90 | 85 | 35.1% |
| [1] | Batch1 G120 | 119 | 39.0% |
| [1] | Batch2 G10 | 9 | 10.7% |
| [1] | Batch2 G30 | 29 | 33.3% |
| [1] | Batch2 G60 | 59 | 21.7% |
| [1] | Batch2 G90 | 85 | 26.3% |
| [1] | Batch2 G120 | 119 | 35.0% |
| [1] | Batch3 G10 | 9 | 56.3% |
| [1] | Batch3 G30 | 29 | 67.5% |
| [1] | Batch3 G60 | 59 | 90.0% |
| [1] | Batch3 G90 | 85 | 81.3% |
| [1] | Batch3 G120 | 119 | 82.5% |
| [1] | Batch4 G10 | 9 | 33.4% |
| [1] | Batch4 G30 | 29 | 28.8% |
| [1] | Batch4 G60 | 59 | 51.0% |
| [1] | Batch4 G90 | 85 | 51.1% |
| [1] | Batch4 G120 | 119 | 79.8% |
| [2] | 70 degree-days | 10 | 33.0% |
| [2] | 140 degree-days | 23 | 35.0% |
| [2] | 210 degree-days | 37 | 41.0% |
| [2] | 280 degree-days | 51 | 43.0% |
| [2] | 350 degree-days | 65 | 54.0% |
| [3] | G20 | 0 | 18.0% |
| [3] | G15-5 | 7 | 37.6% |
| [3] | G15-10 | 34 | 47.1% |
| [3] | G15 | 60 | 66.1% |
| [4] | NW 21-1151 | 15 | 31.0% |
| [4] | NW 13-1151/17-1151/21-5595 | 55 | 51.0% |
| [4] | SE 21-1151 | 15 | 8.0% |
| [4] | SE 13-1151/17-1151/21-5595 | 55 | 33.7% |
| [5] | G20 | 0 | 26.3% |
| [5] | G15 | 68 | 69.0% |
| [5] | G13 | 87 | 73.0% |
| [6] | High temperature | 0 | 69.0% |
| [6] | Natural temperature | 43 | 88.0% |
| [7] | LT | 341 | 8.0% |
| [7] | HT | 11 | 33.0% |
| [8] | 15°C | 61 | 78.0% |
| [8] | 20°C | 0 | 29.0% |
| Table S2 (continued) | | | |
| reference | Treatment name | dph T°<17°C | Percent females |
| [9] | sire a LT | 60 | 11.9% |
| [9] | sire a HT | 7 | 3.3% |
| [9] | sire b LT | 60 | 25.2% |
| [9] | sire b HT | 7 | 12.9% |
| [9] | sire c LT | 60 | 35.0% |
| [9] | sire c HT | 7 | 27.7% |
| [9] | sire d LT | 60 | 48.3% |
| [9] | sire d HT | 7 | 26.6% |
| present study | 32 | 32 | 25.6% |
| present study | 53 | 53 | 44.3% |
| present study | 74 | 74 | 47.1% |
| present study | 90 | 90 | 36.4% |
| present study | 104 | 104 | 29.1% |
| present study | 118 | 118 | 28.2% |
| present study | 132 | 132 | 29.5% |
| present study | 146 | 146 | 32.4% |
| present study | 160 | 160 | 22.2% |
| present study | 174 | 174 | 22.0% |
| present study | 188 | 188 | 15.2% |
| present study | 202 | 202 | 15.2% |
| present study | 216 | 216 | 15.5% |
| present study | 230 | 230 | 8.6% |
| present study | 244 | 244 | 10.7% |

**
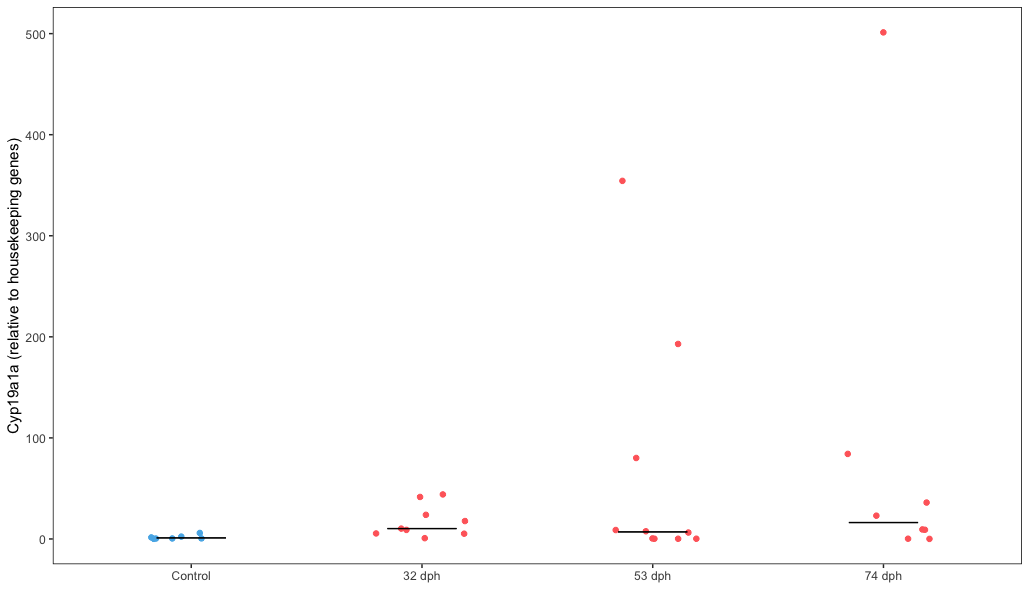
**

**Figure S4:** Aromatase relative expression to housekeeping genes at 96 dph in four distincts conditions, each point represent one individual. In blue, those that were kept at 16°C throughout the experiment, and in red, those that have been transferred at 21°C at each different time point. The black line represent the median.

**References**

1. Navarro-Martín L, Blázquez M, Viñas J, Joly SS, Piferrer F. 2009 Balancing the effects of rearing at low temperature during early development on sex ratios, growth and maturation in the European sea bass (Dicentrarchus labrax). *Aquaculture* **296**, 347–358. (doi:10.1016/j.aquaculture.2009.07.022)

2. Saillant E, Fostier A, Haffray P, Menu B, Thimonier J, Chatain B. 2002 Temperature effects and genotype-temperature interactions on sex determination in the European sea bass (Dicentrarchus labrax L.). *J. Exp. Zool.* **292**, 494–505. (doi:10.1002/jez.10071)

3. Chatain B. 2001 Mise au point d’un process de contrôle du sexe-ratio par la température dans les élevages de bar Dicentrarchus labrax.

4. Koumoundouros G, Pavlidis M, Anezaki L, Kokkari C, Sterioti A, Divanach P, Kentouri M. 2002 Temperature sex setermination in the European sea bass, Dicentrarchus labrax (L., 1758) (Teleostei, Perciformes, Moronidae): critical sensitive ontogenetic phase. *J. Exp. Zool.* **292**, 573–579.

5. Mylonas CC *et al.* 2005 Influence of rearing temperature during the larval and nursery periods on growth and sex differentiation in two Mediterranean strains of Dicentrarchus labrax. *J. Fish Biol.* **67**, 652–668. (doi:10.1111/j.1095-8649.2005.00766.x)

6. Pavlidis M, Koumoundouros G, Sterioti A, Somarakis S, Divanach P, Kentouri M. 2000 Evidence of temperature-dependent sex determination in the European sea bass (Dicentrarchus labrax L.). *J. Exp. Zool.* **287**, 225–232.

7. Abdel I, Abellan E, Alaya M, Garcia-Alcazar S, Lopez-Albors O, Garcia-Alcazar A. 2003 Datos biométricos y de composicion en lubina de tamaño comercial(Dicentrarchus labrax L.) sometida a diferentes temperaturas de cultuivo. In *Libro de Actas del IX Congreso Nacional de Acuicultura*, pp. 333–336. Cadiz, Spain.

8. Sfakianakis DG, Papadakis IE, Papadaki M, Sigelaki I, Mylonas CC. 2013 Influence of rearing temperature during early life on sex differentiation, haemal lordosis and subsequent growth during the whole production cycle in European sea bass Dicentrarchus labrax. *Aquaculture* **412–413**, 179–185. (doi:10.1016/j.aquaculture.2013.07.033)

9. Anastasiadi D, Vandeputte M, Sánchez-Baizán N, Allal F, Piferrer F. 2018 Dynamic epimarks in sex-related genes predict gonad phenotype in the European sea bass , a fish with mixed genetic and environmental sex determination. *Epigenetics* **13**, 988–1011. (doi:10.1080/15592294.2018.1529504)
